# The First Year of *Neurology: Education*: A Look Back at Must-Read Articles, Top Picks, Reviewer Recognition, and What Is Next

**DOI:** 10.1212/NE9.0000000000200075

**Published:** 2023-06-06

**Authors:** Roy Strowd

**Affiliations:** From the Department of Neurology, Wake Forest School of Medicine, Winston Salem, NC.

It has been a little over 1 year since the launch of *Neurology*® *Education* in the spring of 2022. Over the past year, the journal has witnessed tremendous growth, interest from authors, engagement from reviewers, and achieved important milestones.

The journal first launched in April 2022 at the time of the American Academy of Neurology annual meeting. The first article was accepted for publication in July and published in the journal's September issue. This article on an innovative faculty development program for improving narrative comments in the neurology clerkship emphasizes 1 goal for the journal—to disseminate evidence-based approaches to medical education in a way that educators in the clinical neurosciences can enhance their teaching.^1^

Over the first 10 months, the journal received 146 submissions from 12 countries. Of these, 65% were research articles in both the Education Research and Curriculum Innovations categories. Nonresearch submissions included Reviews in Medical Education, Viewpoints, Tweetable Teaching Images, and History of Neurologic and Medical Education papers. The journal's acceptance rate has been 40% with an average turnaround time of 32 days from initial submission. We are off to a great start.

In addition to peer-reviewed submissions, the journal has recognized the value of providing a forum for dialogue between educators, recognition of teaching giants, dissemination of preliminary work, and listening the perspective of the learners. The journal has received more than 30 commentaries and editorials to its blog site. Accepted blog submissions undergo editorial review and rapid dissemination through the journal's website and social media channels. A series on the Learner Perspective provides an outlet for commentary from learners who have participated in the education research projects published in the journal. For example, what is it like to learn shared decision-making and longitudinal outpatient neurology through a patient-centered didactic noon conference? Dr. Mary O'Neal, Dr. Amalie Chen, and patient Frank Modica describe their perspective as a faculty, resident, and patient in this innovative noon conference.^2^ Educators who are interested in the work published in the journal can use these learner perspectives to gain a deeper understanding of how an educational intervention could be implemented in their context.^3^

## Must-Read Articles in 2022–2023

What are the must read articles over the past year in *Neurology: Education*? The top 5 most downloaded articles were:

- *The Inappropriate Consult: Discordant Expectations of Specialty Expertise and Areas for Improvement in Interdisciplinary Resident Education* by Sanky et al.^4^
- *Pictorial Review of Lesion Localization for Patients With Stroke, Upper Limb and Lower Limb Pathology* by Lyon^5^
- *Impact of Burnout on Neurology Residents and Research Fellows in Europe* by Di Liberto et al.^6^
- *The Effect of an X + Y Schedule Model on Neurology Residency Training* by Roy et al.^7^
- And the Across the Spectrum editorial from the first issue titled: *The First Issue: Innovations in Teaching, Assessment, and Developing a Robust Educational Pipeline in Clinical Neuroscience*^8^

Congratulations to these authors on their important scholarship.

## Editor's Top Pick Articles in 2022–2023

The journal has received submissions in each of the many manuscript categories. The editorial board has reviewed all publications, and although not an easy task, it has identified the following as top pick articles in the past year:

- Education Research: *The Effect of an X + Y Schedule Model on Neurology Residency Training* by Roy et al.^7^
- Curriculum Innovations: *Improving Residents' Knowledge and Interest in Outpatient Neurology Through an Interactive Patient-Centered Didactic Series* by Doughty et al.^3^
- Reviews: *Reviews in Medical Education: Advances in Simulation to Address New Challenges in Neurology* by Albin et al.^9^
- Tweetable Teaching Image: *Pictorial Review of Lesion Localization for Patients With Stroke, Upper Limb and Lower Limb Pathology* by Lyon^5^

Congratulations to these authors who deserve recognition for their work. These articles are advancing the field of medical education in neurology and neuroadjacent fields.

## Thanks to Our Reviewers

Peer review is an integral component of scholarly publishing. The process ensures that the highest-quality, methodologically rigorous, peer-vetted research is disseminated. Peer reviewers are the engines of this work and deserve considerable thanks. They donate their time, effort, and expertise. *Neurology: Education* has received an incredible response to its call for peer reviewers with more than 85 individuals indicating interest in reviewing. In its first 10 months, the journal received peer reviews from over 88 of these unique peer reviewers not including reviews from editorial board members. Although the journal sincerely appreciates all of these reviewers, 10 of them have stood out for their frequent and high-quality reviews. These top reviewers deserve special recognition and praise and include

- Jaleed Gilani, MD
- Peter Jin, MD
- Matthew Stuart Robbins, MD, FAAN
- Elina Zankin, MD
- Anthony Fine, MD
- Alexandra Hovaguimian, MD
- Nuri Jacoby, MD
- Justin Jordan, MD, MPH, FAAN
- Doris Kung, DO, FAAN
- Jorge Patino, MD

## What Is Next for Year 2 of the Journal?

Over the coming year, the journal will continue to welcome submissions in all areas of clinical neuroscience education. The journal is interested in articles that span the entire continuum of education. This includes approaches to early exposure and mentoring for high-school, college, and prehealth students; how to teach neurology to health professions students who will and will not pursue specialization in neurology; and innovations in continuing professional development for neurologic practitioners. Importantly, the journal is not a neurology-specific education journal and is interested in submissions on medical education interventions in neuroadjacent fields such as psychiatry, neurosurgery, neuropsychology, neuroradiology, neuropharmacology, neurologic nursing, and others. Timely and time-independent topics are welcome including equity, diversity, inclusion, preparing trainees for practicing medicine within an era of climate change, neuroethics, and artificial intelligence.

## In this Issue of *Neurology: Education*

This issue is divided into educational initiatives for undergraduate medical education, followed by graduate medical education. The first 2 articles focus on ethics and death by neurologic criteria. Ethical dilemmas are frequently encountered in patients with neurologic disorders and may be experienced by neurology and nonneurology providers. These articles wrestle with the meaning of death and how to prepare trainees to address ethics in practice.^10,11^ The remainder of the issue is dedicated to residency and fellowship training covering topics of diversity^12^ and exposure to subspecialty neurology^13^ as well as varying instructional designs including simulation^14^ and near-peer learning.^15^

It has been a year of firsts for *Neurology: Education*. Happy reading.
